# TLR signals posttranscriptionally regulate the cytokine trafficking mediator sortilin

**DOI:** 10.1038/srep26566

**Published:** 2016-05-25

**Authors:** Toshiki Yabe-Wada, Shintaro Matsuba, Kazuya Takeda, Tetsuya Sato, Mikita Suyama, Yasuyuki Ohkawa, Toshiyuki Takai, Haifeng Shi, Caroline C. Philpott, Akira Nakamura

**Affiliations:** 1 Department of Immunology, Kanazawa Medical University, Kahoku Uchinada, Ishikawa, 920-0293, JAPAN; 2 Liver Diseases Branch, National Institute of Diabetes and Digestive and Kidney Diseases, National Institutes of Health, Bethesda, MD 20892, USA; 3 Division of Bioinformatics, Medical Institute of Bioregulation, Kyushu University, Fukuoka 812-8582, Japan; 4 Department of Advanced Medical Initiatives, Faculty of Medicine, Kyushu University, Fukuoka 812-8582, Japan; 5 Department of Experimental Immunology, Institute of Development, Aging and Cancer, Tohoku University, 4-1 Seiryo, Sendai 980-8575, Japan

## Abstract

Regulating the transcription, translation and secretion of cytokines is crucial for controlling the appropriate balance of inflammation. Here we report that the sorting receptor sortilin plays a key role in cytokine production. We observed interactions of sortilin with multiple cytokines including IFN-α, and sortilin depletion in plasmacytoid dendritic cells (pDCs) led to a reduction of IFN-α secretion, suggesting a pivotal role of sortilin in the exocytic trafficking of IFN-α in pDCs. Moreover, sortilin mRNA was degraded posttranscriptionally upon stimulation with various TLR ligands. Poly-rC-binding protein 1 (PCBP1) recognized the C-rich element (CRE) in the 3′ UTR of sortilin mRNA, and depletion of PCBP1 enhanced the degradation of sortilin transcripts, suggesting that PCBP1 can act as a *trans*-acting factor to stabilize sortilin transcripts. The nucleotide-binding ability of PCBP1 was impaired by zinc ions and alterations of intracellular zinc affect sortilin expression. PCBP1 may therefore control the stability of sortilin transcripts by sensing intracellular zinc levels. Collectively, our findings provide insights into the posttranslational regulation of cytokine production through the posttranscriptional control of sortilin expression by TLR signals.

Infection by various pathogens and subsequent cellular stimuli leads to rapid inflammatory responses through a set of pattern-recognition receptors (PRRs) that includes Toll-like receptors (TLRs), helicase RIG-I–like receptors and biosensor Nod–like receptors^1^. The PRR signals induce production of proinflammatory cytokines, such as tumor necrosis factor (TNF) and interleukin 6 (IL-6), as well as type I interferons (IFNs) and chemokines, and type I IFNs and cytokines are involved in the inflammation process. Excessive immune responses are defined by overproduction of cytokines and contribute to tissue damage by chronic inflammation as well as the development of autoimmune diseases. Therefore, it is imperative to maintain cytokines at resting-state levels by tight control of the signaling pathways that govern cytokine production.

Regulating the secretion of cytokines from immune cells, as well as their expression, is important for the suppression of excessive immune responses. Although many studies have elucidated the molecular details of signaling pathways for cytokine production^1^, we know little about the mechanisms of cytokine secretion from immune cells^2^.

Sortilin is a type 1 membrane protein and is evolutionarily conserved from yeast to humans^3^. It is a multifunctional receptor that binds many kinds of ligands, and thus exerts diverse cellular functions such as lipoprotein metabolism and insulin-regulated glucose uptake^3^,^4^,^5^,^6^. Sortilin is predominantly found in early endosomes and in the trans-Golgi network^7^, and its localization is regulated by cargo adaptor complexes^3^. Recent studies have demonstrated that loss of sortilin impairs the secretion of IL-6 and IFN-γ cytokines in macrophages and T cells^8^,^9^. Interestingly, surface plasmon resonance (SPR) analysis and immunofluorescence microscopy have indicated a physiological interaction between sortilin and IL-6 and IFN-γ^8^,^9^, and sortilin deficiency in immune cells leads to the attenuation of inflammation and atherosclerosis^8^. These observations indicate that IL-6 and IFN-γ are sortilin ligands, and that sortilin plays a key role in cytokine production. However, it remains unclear whether sortilin is able to recognize other immune cytokines, and how its expression is regulated upon stimulation in immune cells.

Recent research has demonstrated that *trans*-acting factors such as RNA-binding proteins (RBPs) play a key role in the posttranscriptional regulation of immunity-related mRNAs^10^, and this regulation is also important for the repression of excessive immune responses, such as chronic inflammation and autoimmune diseases^11^. Specific *cis*-elements in the 3′ UTR, such as AU-rich elements (AREs) and stem-loop structures, are possessed by many immunity-related mRNAs and contribute to their degradation or stabilization, and various *trans*-acting factors involved in this regulation have also been identified^10^,^12^. Tristetraprolin, AUF1 and HuR are ARE-binding proteins that control the stability of mRNAs containing AREs, and stem-loop structures are recognized by Roquin and Regnase-1 followed by destabilization of target mRNAs^10^,^11^,^12^. The functions of these *trans*-acting factors are regulated by a number of kinase pathways, resulting in the coordinated expression of their target mRNAs^10^,^11^.

Poly-rC-binding protein (PCBP) 1 (also called hnRNP E1, or alpha-CP-1) is a multifunctional RBP that recognizes C-rich elements (CREs) of single-stranded RNA and has diverse functions, such as in RNA processing, translation and stability, through its binding to CREs^13^,^14^. A recent study using knockout mice has demonstrated that disruption of PCBP1 causes embryonic lethality at the peri-implantation stage^15^, indicating that PCBP1 is essential for mouse embryonic development. PCBP1 is also crucial for the metabolism of the transition metal iron, acting as an iron chaperone^16^,^17^,^18^,^19^. PCBP2, a paralog of PCBP1, has been reported to regulate microRNA processing by modulating its association with Dicer depending on cytosolic iron status^20^, suggesting that PCBP2 regulates RNA processing by sensing cellular iron status. However, it is unknown whether PCBP1 can bind transition metals besides iron, and whether the binding of iron or other metals to PCBP1 alters its nucleotide-binding ability.

Transition metals including iron and zinc are known to have crucial roles in innate immune responses^21^,^22^,^23^,^24^. For example, TLR4 signals produce hepcidin, a key regulator of iron metabolism, for the iron sequestration response, which is postulated to combat infections by limiting iron availability to microbes^25^, and intracellular zinc functions as a signaling factor in immune responses^21^. Although PCBP1 and PCBP2 are reported to contribute to antiviral responses by inhibiting mitochondrial antiviral signaling in innate immunity^26^,^27^, little is known about the functional roles of the nucleotide- and metal-binding abilities of PCBP1 and PCBP2 in innate immunity.

Here, we demonstrate by SPR analysis that sortilin interacts with various cytokines as well as IL-6 and IFN-γ, and is involved in the transport of IFN-α in plasmacytoid dendritic cells (pDCs), which are known as type I IFN-producing cells and are indispensable for antiviral immune responses^28^. Notably, we observed that the expression of sortilin was negatively regulated by TLR signals at the posttranscriptional level. Interestingly, the 3′ UTR of sortilin mRNA contains a CRE, a structure which contributes to the stability of mRNA as a *cis*-element^13^,^14^. We describe here the interaction of PCBP1 with the CRE in the 3′ UTR of sortilin transcripts, and the involvement of PCBP1 in the stabilization of sortilin mRNA. Moreover, we show that the transition metal zinc interferes with the nucleotide binding of PCBP1 to the CRE and that intracellular zinc status affects sortilin expression. Our observations provide insights into the posttranslational regulation of cytokine production via the posttranscriptional control of sortilin expression.

## Results

### Sortilin interacts with various cytokines

**Figure 1:**
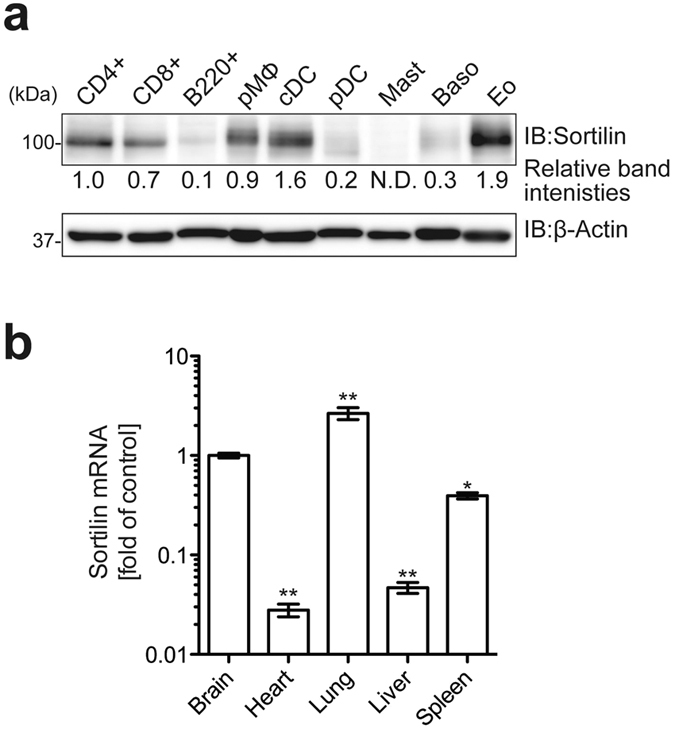
Expression profiles of Sortilin in immune cells and tissues. (**a**) Immunoblot analysis of sortilin in various immune cells. CD4^+^, CD8^+^ and B220^+^ cells were directly isolated from splenic cells by MACS. Macrophages were isolated from the peritoneal cavity, and other cells were derived from bone marrow cells. Whole cell lysates were separated by SDS-PAGE and sortilin expression was determined by immunoblot analysis. β-Actin was used as an internal control. CD4^+^, splenic CD4^+^ cells; CD8^+^, splenic CD8^+^ cells; B220^+^, splenic B220^+^ cells; pMØ, peritoneal macrophages; cDC, bone marrow-derived conventional DCs; pDC, bone marrow-derived plasmacytoid DCs; Mast, bone marrow-derived mast cells; Baso, bone marrow-derived basophils; Eo, bone marrow-derived eosinophils. Relative band intensities of sortilin compared to CD4^+^ are shown. (**b**) Sortilin expression in various tissues. Total RNA was isolated from mouse tissues and sortilin mRNA was quantified by qRT-PCR. GAPDH was used as an internal control for normalization. Data are mean ± SD (n = 3). Asterisks denote the statistical difference from brain. *p < 0.05 **p < 0.01.

To investigate sortilin functions in immune cells, we initially characterized gene expression of sortilin in various immune cells and mouse tissues by immunoblot analysis and quantitative real time PCR (qRT-PCR), respectively. Immunoblot analysis revealed that sortilin was predominantly expressed in CD4^+^ and CD8^+^ splenocytes, and peritoneal macrophages. Bone marrow-derived conventional dendritic cells (cDCs) and eosinophils also displayed higher expression levels of Sortilin (Fig. 1a). B220^+^ splenocytes and bone marrow-derived plasmacytoid DCs (pDCs) and basophils showed lower expression levels of sortilin, while sortilin was hardly detected in bone marrow-derived mast cells (Fig. 1a). qRT-PCR analysis revealed that sortilin was predominantly expressed in brain, lung and spleen, whereas lower expression of sortilin was observed in heart and liver (Fig. 1b). These data indicate that the expression levels of sortilin differ in various immune cells and tissues.

**Figure 2:**
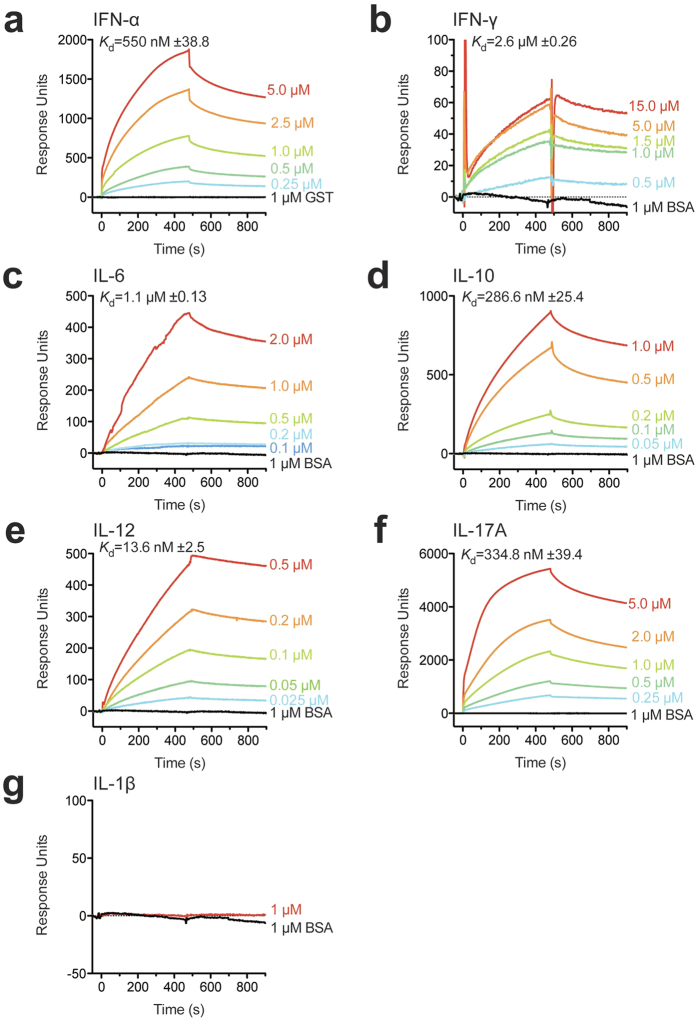
Sortilin interacts with various cytokines. The binding of cytokines to sortilin was measured by surface plasmon resonance analysis. Cytokine binding to immobilized recombinant sortilin was measured with a Biacore X instrument. Sensorgrams of surface plasmon resonance were obtained with various concentrations of recombinant IFN-α (**a)**, IFN-γ (**b**), IL-6 (**c**), IL-10 (**d**), IL-12 (**e**), IL-17A (**f**) and IL-1β (**g**). BSA or GST was used as a negative control. All curves indicate the response after subtraction of non-specific binding, which was determined as the binding of analytes to a BSA-coated flow cell. The mean Kd values ± SEM (n = 3) are shown.

A recent study has reported that sortilin deficiency leads to a decrease of IFN-γ and IL-6 secretion, and an interaction of sortilin with these cytokines was observed by surface plasmon resonance (SPR) analysis^8^, suggesting that sortilin is involved in exocytic trafficking of cytokines. Sortilin is known to bind various proteins that are directed to intracellular trafficking pathways^3^,^4^,^5^,^6^, raising the possibility that sortilin participates in the trafficking of various other cytokines in addition to IFN-γ and IL-6. To investigate whether sortilin can bind other cytokines, we performed SPR analysis with recombinant mouse sortilin and cytokines. We confirmed that sortilin interacts with IFN-γ and IL-6 (Fig. 2b,c). Additionally, we observed an interaction of sortilin with IFN-α, IL-10, IL-12 and IL-17A (Fig. 2a,d–f), but not with IL-1β (Fig. 2g). These data suggest that sortilin is involved in exocytic trafficking of IFN-α, IL-10, IL-12 and IL-17A, as well as IFN-γ and IL-6, in immune cells.

### Sortilin is involved in IFN-α secretion from pDCs

**Figure 3:**
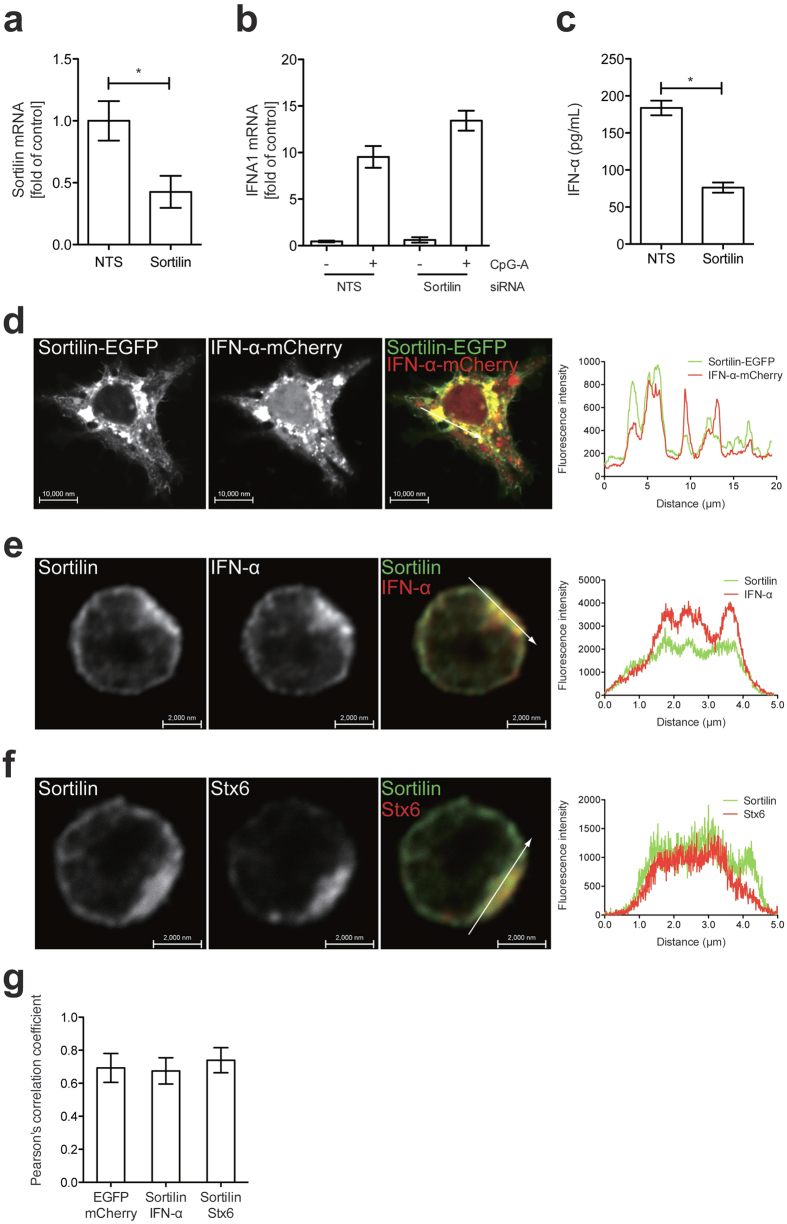
Sortilin is involved in IFN-α secretion in pDCs. (**a**) Confirmation by qRT-PCR of siRNA knockdown efficiency of sortilin in pDCs. Data are mean ± SD (n = 3). *p < 0.01. (**b**) Quantification of IFNA1 mRNA after sortilin knockdown. Sortilin siRNA or non-targeting sequence (NTS) siRNA (both 300 nM) was introduced into pDCs by nucleofection for 24 h. Total RNA was isolated after 6 h of incubation with CpG-A, and IFNA1 mRNA was quantified by qRT-PCR. Data are mean ± SD (n = 3). (**c**) Quantification of IFN-α secretion by ELISA. Sortilin siRNA or NTS siRNA (both 300 nM) was introduced into pDCs by nucleofection for 24 h. The cell culture supernatant was collected after 18 h of incubation with CpG-A, and secreted IFN-α was quantified by ELISA. Data are mean ± SD (n = 4). *p < 0.01. (**d**) Confocal microscopy of sortilin-EGFP and IFN-α-mCherry in HEK293T cells. Plasmids pEGFP-Sortilin and pmCherry-IFNA2 were simultaneously introduced into HEK293T cells. (**e**) Confocal microscopy of sortilin and IFN-α in pDCs. pDCs were stimulated with 3 μM of CpG-A for induction of IFN-α. Endogenous sortilin and IFN-α were detected by indirect immunofluorescence analysis with specific antibodies. (**f**) Confocal microscopy of sortilin and syntaxin 6 (Stx6) in pDCs. Endogenous sortilin and Stx6 were detected by indirect immunofluorescence analysis with specific antibodies. All microscopic images were obtained with a laser scanning microscope and analyzed with ZEN 2010 software (Carl Zeiss). The linescan analysis (right panels of **d**–**f**) was performed with ZEN2010 software (Carl Zeiss) White arrows represent the direction and distance of the track of the linescan. (**g**) Pearson’s correlation coefficients of images of sortilin-EGFP and IFN-α-mCherry in HEK293T cells (left), sortilin (DyLight 488) and IFN-α (DyLight 649) in pDCs (middle) or sortilin (DyLight 488) and Stx6 (DyLight 649) in pDCs (right). Data are mean ± SD (n = 30).

pDCs are known to be a major source of type I IFN in response to viruses that are recognized by Toll-like receptor 7 (TLR7) and TLR9. The expression of sortilin in pDCs (Fig. 1a) and the physiological interaction of sortilin with IFN-α (Fig. 2a) suggested the involvement of sortilin in exocytic trafficking of IFN-α. To address this possibility, we depleted sortilin with siRNA, and then assessed IFN-α production in pDCs by qRT-PCR and ELISA. We confirmed the knockdown efficiency of sortilin in pDCs by nucleofection and qRT-PCR (Fig. 3a). ELISA analysis indicated that upon stimulation with CpG-A, a TLR9 ligand, IFN-α production was substantially decreased in sortilin-depleted cells (Fig. 3c). qRT-PCR analysis revealed that induction of IFNA1 transcription upon CpG-A stimulation was not affected by sortilin depletion (Fig. 3b), indicating that TLR9 signals for IFNA1 transcription remained intact despite sortilin knockdown. These observations suggested that sortilin knockdown impaired the exocytic pathway of IFN-α but not TLR9 signals for IFNA1 induction.

To investigate whether sortilin and IFN-α interacted in cells, we initially constructed vectors encoding EGFP and mCherry fused to the C termini of sortilin and IFN-α, respectively, and captured fluorescent signals in transiently transfected HEK293T cells for simple detection of both proteins on microscopic analysis. Confocal microscopic imaging demonstrated that fluorescent signals from EGFP overlapped well with those from mCherry (Fig. 3d,g), indicating that sortilin-EGFP co-localized with IFN-α-mCherry in these cells. Next, to investigate whether sortilin could directly associate with IFN-α in pDCs, we observed co-localization of endogenous sortilin with endogenous IFN-α by indirect immunofluorescence analysis with specific antibodies. Confocal microscopic analysis indicated co-localization of endogenous sortilin with endogenous IFN-α (Fig. 3e,g), indicating that sortilin co-localized with IFN-α in pDCs. In addition, we confirmed co-localization of endogenous sortilin with syntaxin 6 (Stx6), a TGN marker, in pDCs as previously reported^29^ (Fig. 3f,g). The distribution of transfected sortilin-EGFP and IFN-α-mCherry in HEK293T cells differs slightly from that of endogenous sortilin and IFN-α in pDCs. This difference is likely due to the very different cell morphologies and nucleo-cytoplasmic ratios in pDCs versus HEK293 cells. The distribution of sortilin and IFN-α within the secretory pathway is also likely to be altered somewhat by overexpression in the HEK293T cells. Taken together, these results suggest that sortilin is indeed involved in exocytic trafficking of IFN-α in pDCs.

### TLR signals negatively regulate sortilin expression

**Figure 4:**
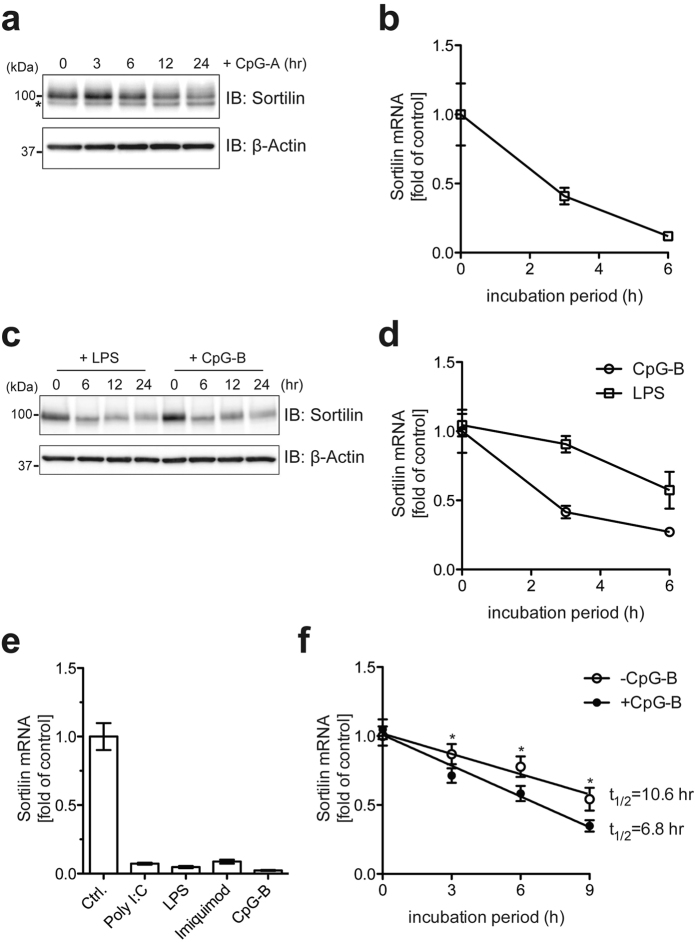
TLR signals lead to posttranscriptional degradation of sortilin. (**a**,**b**) The time course of sortilin degradation following CpG-A (3 μM) stimulation was determined by immunoblotting (**a**) and qRT-PCR (**b**) in pDCs. The asterisk represents a nonspecific band. (**c**,**d**) The time course of sortilin degradation following LPS (0.1 μg/ml) or CpG-B (3 μM) stimulation was determined by immunoblotting (**c**) and qRT-PCR (**d**) in macrophages. Data are mean ± SD (n = 3). (**e**) Quantification of sortilin mRNA after stimulation with various TLR ligands in macrophages. Macrophages were stimulated with poly I:C (10 μg/ml), LPS (1 μg/ml), imiquimod (10 μg/ml) and CpG-B (3 μM) for 6 h, followed by quantification of sortilin mRNA by qRT-PCR. Data are mean ± SD (n = 3). (**f**) Posttranscriptional degradation of sortilin mRNA. Sortilin mRNA was quantified by qRT-PCR after CpG-B stimulation (1 μM) of cells in medium containing 1 μg/ml ActD. Linear regression analyses were performed with GraphPad Prism software to determine the half-life of sortilin mRNA. Data are mean ± SD (n = 3). *p < 0.05.

We noted that stimulation with CpG-A led to loss of endogenous sortilin mRNA and protein in pDCs (Fig. 4a,b). To investigate whether this phenomenon also occurred in other cells, macrophages were stimulated with the TLR ligands LPS (TLR4) and CpG-B (TLR9), followed by quantification of sortilin mRNA and protein. qRT-PCR and immunoblot analysis indicated that stimulation by both LPS and CpG-B resulted in the disappearance of sortilin mRNA and protein (Fig. 4c,d). qRT-PCR analysis also revealed that sortilin was decreased upon stimulation with poly I:C (TLR3) and imiquimod (TLR7) as well as with LPS and CpG-B (Fig. 4e). These results suggest that TLR signals negatively regulate the expression of sortilin. Next, to investigate whether sortilin expression was regulated at the transcriptional level, we monitored sortilin expression upon CpG-B stimulation when transcription was repressed by actinomycin D (ActD). qRT-PCR analysis indicated that degradation of sortilin mRNA was facilitated in the presence of CpG-B (Fig. 4f). Linear regression analysis revealed a difference in sortilin mRNA half-life between CpG-B-stimulated and unstimulated cells (Fig. 4f), indicating that degradation of sortilin mRNA upon TLR stimulation occurred at a posttranscriptional step. These results suggest that sortilin expression is regulated posttranscriptionally by TLR signals.

### PCBP1 is involved in stabilization of sortilin mRNA

**Figure 5:**
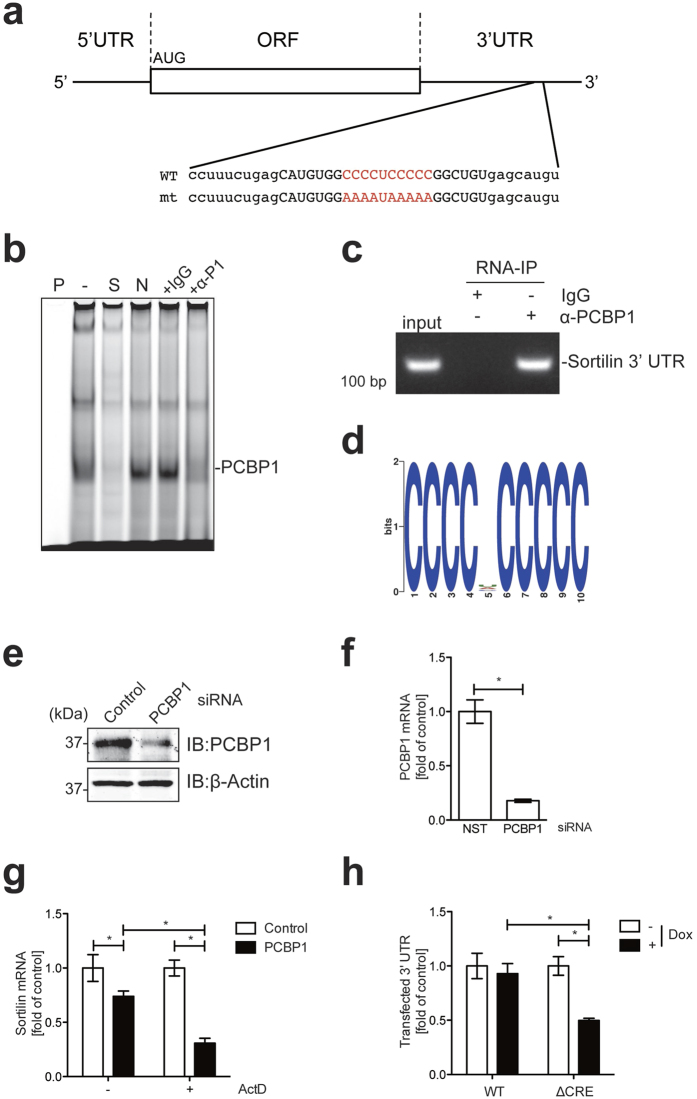
PCBP1 binds to C-rich elements in the 3′UTR of sortilin mRNA. (**a**) Scheme of mouse sortilin mRNA containing defined mutations of the PCBP1-binding site. Only the sequence around the CRE in the 3′ UTR is shown. Synthetic RNA probes for RNA EMSA are shown as upper-case letters and CREs are in red. (**b**) RNA EMSA analysis. An IR800-conjugated WT CRE probe was mixed with binding buffer alone (P) or whole cell lysates from macrophages (−). Samples were separated by PAGE. Five-fold molar excess amounts of unlabeled WT CRE and mt CRE were added as specific competitor (S) and nonspecific competitor (N), respectively. Antibody supershift experiments were performed with anti-PCBP1 antibody (+α-P1). Rabbit IgG (+IgG) was used as a negative control in antibody supershift experiments. (**c**) RNA immunoprecipitation (RIP) analysis of PCBP1. RT-PCR was performed with RNA immunoprecipitated by anti-PCBP1 antibody to amplify the CRE-containing region of the 3′ UTR of sortilin mRNA. RNA immunoprecipitated by rabbit IgG (IgG) was used as a negative control. (**d**) Depiction of sequence significantly enriched by RIP-seq analysis as a position weight matrix (E-value  =  7.8e-80). (**e**,**f**) Depletion of PCBP1 in RAW264.7 cells. Cells were transfected with siRNAs against PCBP1 or with a control non-targeting siRNA, and were harvested 2 d after transfection. Cell lysates were subjected to immunoblotting (**e**) with the indicated antibodies, and total RNAs were subjected to qRT-PCR (**f**) to quantify PCBP1 mRNA. Data are mean ± SD (n = 3). *p < 0.01. (**g**) Destabilization of sortilin mRNA by PCBP1 depletion in cells. RAW264.7 cells were transfected with control or PCBP1 siRNA for 48 h and then treated with 1 μg/ml ActD for 9 h. Total RNAs were subjected to qRT-PCR to quantify sortilin mRNA. Data are mean ± SD (n = 3). *p < 0.01. (**h**) Destabilization of the 3′ UTR lacking the CRE. RAW264.7 cells were co-transfected with pTet-off and either pTRE2-WT or pTRE2-ΔCRE for 18 h, and then treated with 1 μg/ml of doxycycline (Dox) for 2 h. Total RNAs were subjected to qRT-PCR to quantify the 3′ UTR mRNA. Data are mean ± SD (n = 3). *p < 0.01.

RBPs are known to be involved in splicing, export, stability, localization and translation of mRNA, and in almost all cases they recognize *cis*-elements located in the 3′ and/or 5′ UTRs. In immune responses, RBPs also play an important role in posttranscriptional regulation of immunity-related mRNA^10^. To elucidate the posttranscriptional control mechanisms of sortilin mRNA, we searched for typical elements in the UTRs of sortilin mRNA that might be involved in mRNA stability, and found a C-rich element (CRE) in the 3′ UTR (CCCCUCCCCC sequence) (Fig. 5a). Poly-rC-binding protein (PCBP) can bind CREs in single-stranded RNA and has various roles such as stability, processing and translational regulation of RNA^14^. To investigate whether PCBP1 binds to the CRE in the 3′ UTR of sortilin mRNA, we carried out an RNA electromobility shift assay (EMSA) with cell lysate. When cell lysate prepared from macrophages was incubated with an RNA probe containing the CRE, shifted bands were observed, and these bands were diminished by incubation with an excess amount of unlabeled wild-type probe (Fig. 5b, lane S). The addition of an excess amount of mutant probe (mt CRE: Fig. 5a) did not interfere with the PCBP1-RNA interaction, indicating that formation of the observed protein-RNA complex depended on the CRE. Furthermore, the band was largely supershifted by incubation of cell lysate with anti-PCBP1 antibody (Fig. 5b), indicating that the observed protein-RNA complex mostly contained PCBP1.

We next performed RNA-immunoprecipitation (RIP) analysis to investigate whether PCBP1 interacted with the 3′ UTR of endogenous sortilin mRNA. We found that the CRE-containing sequence in the 3′ UTR of sortilin mRNA was amplified in the samples immunoprecipitated with anti-PCBP1 (Fig. 5c), indicating that PCBP1 can interact with the CRE in the 3′ UTR of endogenous sortilin mRNA. Furthermore, we carried out RIP-seq analysis to identify mRNAs that were recognized by PCBP1 and to define elements bound by PCBP1. Motif analysis revealed significant enrichment of a CCCCNCCCCC motif in the RIP sample (Fig. 5d), indicating that PCBP1 bound to mRNAs which possessed this motif. We found that, in addition to sortilin, various transcripts possessing CREs in their 3′ UTR co-precipitated with PCBP1 (Supplementary Table II), although gene ontology analysis indicated no particular functional bias among the genes identified by our RIP-seq analysis.

We next assessed PCBP1 depletion with siRNA to investigate whether PCBP1 was involved in stabilizing sortilin mRNA. We confirmed that PCBP1 was efficiently decreased in siRNA-transfected cells at the protein and mRNA levels (Fig. 5e,f). Inhibition of transcription by ActD accelerated degradation of sortilin mRNA in PCBP1-knockdown cells, as revealed by qRT-PCR (Fig. 5g), indicating that depletion of PCBP1 led to the destabilization of sortilin mRNA. Furthermore, we assessed the contribution of the CRE to the stability of sortilin mRNA. To do this, we cloned the CRE-containing sequence in the 3′ UTR of sortilin with or without the CCCCUCCCCC sequence into the vector pTRE2 for the control of transcription activity with doxycycline, and investigated whether the loss of the CRE affected the stability of mRNA using the Tet-Off system. qRT-PCR analysis indicated that disruption of the CRE facilitated degradation of transcripts when transcription was inhibited with doxycycline (Fig. 5h), indicating that the CRE is critical for mRNA stability as a *cis*-element. Taken together, these data suggest that PCBP1 contributes to the stabilization of sortilin transcripts by binding to the CRE in the 3′ UTR of sortilin mRNA.

### Cellular zinc status affects the stabilization of sortilin transcripts

**Figure 6:**
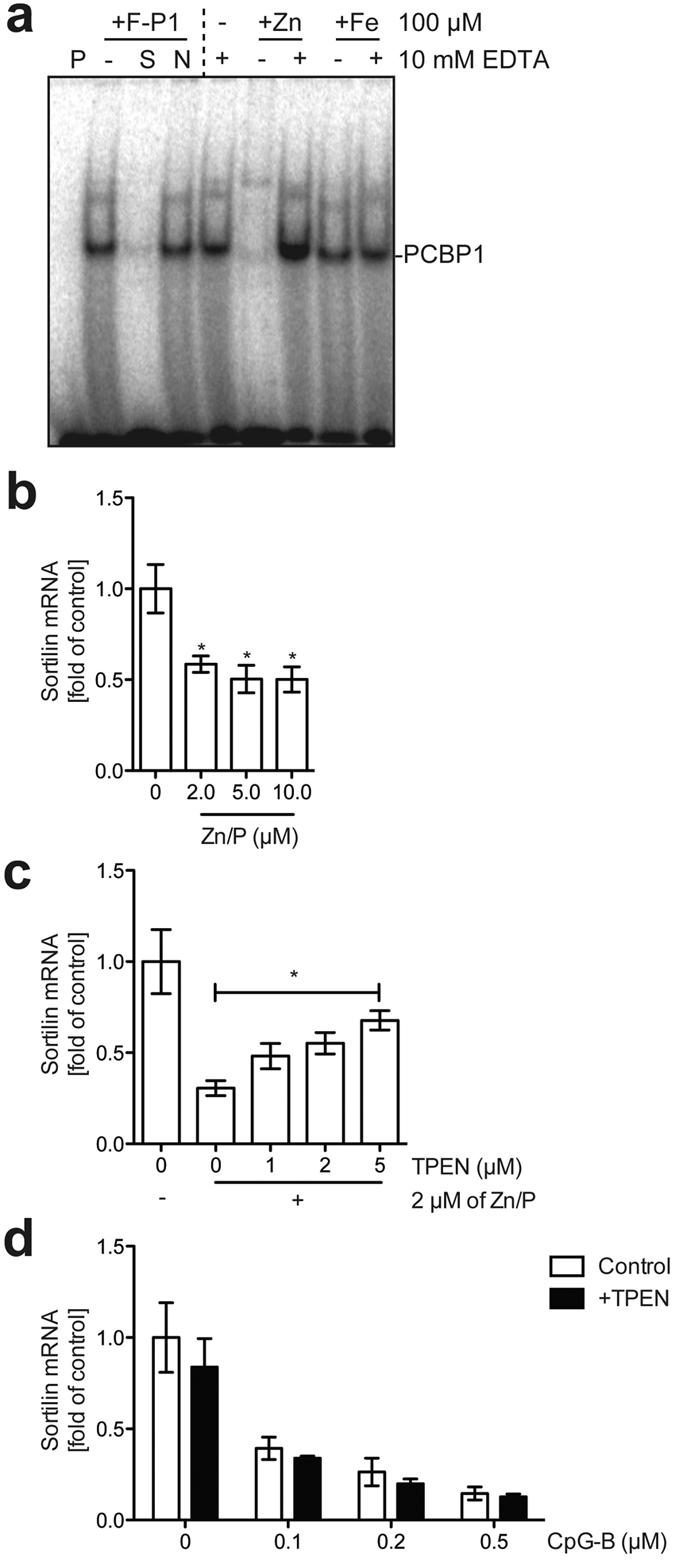
Zinc ions affect the nucleotide-binding ability of PCBP1 and sortilin mRNA stability. (**a**) EMSA analysis of PCBP1. ^32^P-labeled poly-C oligonucleotide probe (100 nM) was mixed with buffer alone (P) or yeast lysates expressing FLAG-PCBP1. Samples were separated by PAGE. A 10-fold molar excess of unlabeled poly-C oligonucleotide and mutated oligonucleotide was added as specific competitor (S) and nonspecific competitor (N), respectively. Zinc sulfate and ferrous ammonium sulfate (both 100 μM) with or without 10 mM EDTA were incubated with samples prior to incubation with the probes. (**b**) Quantification of sortilin mRNA after supplementation with zinc sulfate and the ionophore pyrithione. Macrophages were treated with zinc sulfate and pyrithione (Zn/P) for 6 h and then harvested for isolation of total RNA. Total RNAs were subjected to qRT-PCR to quantify sortilin mRNA. Data are mean ± SD (n = 3). *p < 0.01 versus control. (**c**) Zinc chelation interferes with destabilization of sortilin mRNA by zinc. Macrophages were treated with 2 μM Zn/P with or without various concentrations of TPEN for 6 h. Total RNAs were subjected to qRT-PCR to quantify sortilin mRNA. Data are mean ± SD (n = 3). *p < 0.01. (**d**) Zinc chelation does not prevent CpG-B-dependent Sortilin mRNA degradation. Sortilin mRNA was quantified after 1 μM CpG-B stimulation for 6 h with or without 2 μM TPEN. Data are mean ± SD (n = 3).

PCBP1 also functions as a cytosolic iron chaperone for delivery to various iron-containing enzymes, such as prolyl hydroxylase 2 and deoxyhypusine hydroxylase/monooxygenase, as well as the cytosolic iron storage protein ferritin^30^. A recent study has reported that PCBP2, a paralog of PCBP1, regulates the processing of microRNA by modulating the association of PCBP2 with Dicer depending on cytosolic iron status^20^, although it is unknown whether the nucleotide-binding ability of PCBP1 is comparably modulated by the binding of iron or other transition metals. To investigate if the nucleotide-binding activity of PCBP1 was influenced by transition metal binding, we carried out EMSA analysis with lysates of yeast cells expressing FLAG-PCBP1 in the presence of transition metals. Interestingly, nucleotide binding by PCBP1 was unchanged when the lysate was incubated with iron, but was significantly reduced when the lysate was incubated with zinc (Fig. 6a). Incubation with an excess amount of EDTA canceled the interference by zinc ions with the nucleotide-binding ability of PCBP1 (Fig. 6a), indicating a reversible inhibition of PCBP1 nucleotide-binding ability by zinc.

Because TLR signals induce an intracellular increase in the cytosolic free zinc content^31^,^32^, we postulated that intracellular zinc levels play a key role in the regulation of sortilin mRNA by TLR signals. To address this, we quantified sortilin transcripts in macrophages after the addition of zinc together with the ionophore pyrithione. As we expected, sortilin mRNA was decreased upon supplementation of zinc together with pyrithione (Fig. 6b). Addition of the membrane-permeable zinc chelator TPEN prevented zinc-dependent degradation of sortilin mRNA (Fig. 6c), indicating that intracellular zinc levels affect sortilin expression. From these findings we infer that an increase of intracellular zinc concentration leads to dissociation of PCBP1 from the 3′ UTR of sortilin mRNA, resulting in destabilization of sortilin mRNA. Interestingly, TPEN did not prevent TLR signaling-dependent degradation of sortilin transcripts following CpG stimulation (Fig. 6d), suggesting that other signaling pathways and/or factors also play an important role in sortilin mRNA degradation by TLR signaling.

## Discussion

The PRR signal, which is induced by pathogen infection and cellular stimuli, leads to the production of various cytokines and chemokines by immune cells. Appropriate inflammatory responses to various factors such as microbial infection are important for the protection of the body against detrimental stimuli, whereas excessive immune responses, defined by an overproduction of cytokines, lead to chronic inflammation as well as the development of autoimmune diseases. It is therefore assumed that elaborate mechanisms for cytokine secretion, as well as transcription and translation, exist in immune cells to control appropriate cytokine production.

The multi-ligand type-1 receptor sortilin exerts diverse cellular functions in the nervous system, lipoprotein metabolism and insulin-regulated glucose uptake^3^,^4^,^5^,^6^. Moreover, previous studies have demonstrated that sortilin directly interacts with IL-6 and IFN-γ and is involved in the exocytic trafficking of these cytokines in T cells and macrophages^8^,^9^. In this study, we found that in addition to T cells and macrophages, sortilin was broadly expressed in immune cells (Fig. 1a). Notably, however, mast cells expressed almost no sortilin compared to the other cells tested in this study (Fig. 1a), although they did produce many kinds of cytokines including IL-6 and IFN-γ, suggesting the possible involvement of other factors including paralogs of sortilin in cytokine secretion in mast cells. Our SPR analysis revealed that in addition to IL-6 and IFN-γ, sortilin interacted with IFN-α, IL-10, IL-12 and IL-17A (Fig. 2) but not with IL-1β (Fig. 2g), suggesting that sortilin is not involved in IL-1β secretion. Unlike other cytokines such as IFN-α and IL-6, IL-1β is synthesized as an inactive pro-form in the cytosol; pro-IL-1β is cleaved by caspase-1, followed by the immediate secretion of mature IL-1β from the cell^33^. Since pro-IL-1β does not possess a typical signal sequence for secretion, IL-1β is presumably secreted through a different pathway from the conventional endoplasmic reticulum (ER)-Golgi route^34^. Sortilin predominantly exists in the trans-Golgi network^7^, suggesting that in immune cells it mainly functions for cytokine secretion via the conventional ER-Golgi secretion pathway. IL-10, IL-12 and IL-17A, with which sortilin interacts (Fig. 2d–f), are known to be secreted by regulatory T cells, conventional DCs and Th17 cells, respectively, suggesting the involvement of sortilin in exocytic trafficking of these cytokines. Taken together, these findings imply that sortilin plays a key role in exocytic trafficking of cytokines through the ER-Golgi secretion route in various immune cells.

pDCs are known to sense viruses via TLR7 and TLR9, and to secrete large amounts of type I IFN in response. In addition to the role of pDCs in antiviral immunity, abnormal activation of pDCs is also known to contribute to pathogenesis of autoimmune diseases, such as systemic lupus erythematosus (SLE), psoriasis and type 1 diabetes^28^. In SLE, elevated type I IFN secretion from pDCs contributes to pathogenesis^35^. We found that the depletion of sortilin in pDCs led to a reduction of IFN-α secretion (Fig. 3a–c), and that sortilin could directly interact with IFN-α (Figs 2a and 3d,e). The induction of IFNA1 transcripts upon stimulation with TLR9 ligands remained intact in sortilin-depleted cells (Fig. 3b), indicating that sortilin knockdown impairs IFN-α secretion in pDCs. These observations suggest that sortilin has an important role in exocytic trafficking of IFN-α in pDCs, and we infer that it contributes to immune responses that culminate in viral or autoimmune inflammation. A recent study has shown that reconstitution of mice with sortilin-deficient bone marrow cells leads to a decline in inflammation and atherosclerosis^8^, suggesting the importance of sortilin in the development of inflammation. Further *in vivo* studies are required to elucidate the role of sortilin in inflammation responses.

We observed that sortilin was posttranscriptionally downregulated upon TLR stimulation (Fig. 4), and we suggest that this posttranscriptional control modulates exocytic trafficking of cytokines, resulting in the suppression of cytokine overproduction in immune responses at the posttranslational level. Recent studies have demonstrated that posttranscriptional regulation of immunity-related mRNAs is important for the repression of excessive immune responses^10^,^11^, and various *trans*-acting factors which bind to *cis*-elements in the 3′ UTRs of immunity-related mRNAs are involved in this regulation. We found that the 3′ UTR of sortilin mRNA possesses a CRE as a *cis*-element (Fig. 5a); CREs are known to contribute to the stability of mRNA^13^,^14^. EMSA and RIP analyses indicated that PCBP1 bound to this and other CREs (Fig. 5b–d). Depletion of PCBP1 facilitated the degradation of sortilin mRNA (Fig. 5e–g), and disruption of the CRE destabilized the mRNA (Fig. 5h), indicating that PCBP1 functions as a *trans*-acting factor for the stabilization of sortilin mRNA and that the CRE is critical for its stability. These results suggest both the importance of PCBP1 for sortilin regulation at the posttranscriptional levels, and the involvement of PCBP1 in the regulation of cytokine production via control of sortilin expression. Interestingly, we observed that PCBP1 expression was unchanged following TLR stimulation (data not shown), suggesting that CRE-RNA/PCBP1 dissociation upon TLR stimulation triggers sortilin mRNA degradation. Previous studies have demonstrated that phosphorylated PCBP1 loses its ability to associate with ribonucleotide^36^,^37^, from which one might postulate that TLR-dependent phosphorylation of PCBP1 leads to dissociation of the CRE-RNA/PCBP1 complex, followed by degradation of sortilin mRNA. However, we could not detect phosphorylation of PCBP1 upon TLR9 stimulation in macrophages (data not shown), suggesting that phosphorylation of PCBP1 was not involved in the control of sortilin mRNA stabilization in response to TLR signals.

PCBP1 is an iron chaperone that can bind iron in addition to its function as an RBP^16^,^17^,^18^,^19^, although no relationship between nucleotide- and iron-binding ability has been reported for PCBP1. Moreover, it has not been shown that PCBP1 can bind transition metals other than iron. EMSA analysis indicated that iron did not alter the ability of PCBP1 to bind to nucleotides, whereas zinc did modulate its nucleotide-binding ability (Fig. 6a), suggesting that PCBP1 can bind zinc ions. We also found that the expression level of sortilin was modulated by intracellular zinc level (Fig. 6b,c). Previous studies have demonstrated that TLR signals induce an increase of the cytosolic free zinc content^31^,^32^. Based on these observations, we propose a model in which a TLR-zinc signaling axis triggers dissociation of the CRE-RNA/PCBP1 complex, resulting in the degradation of sortilin mRNA to prevent overproduction of cytokines at the posttranslational step. We infer that PCBP1 may sense intracellular zinc levels to regulate sortilin expression by binding to the CRE in the 3′ UTR of sortilin mRNA, although further experiments are needed to reveal the molecular details of zinc ion-dependent regulation of sortilin via PCBP1.

Meanwhile, we observed that degradation of sortilin mRNA upon TLR stimulation was not affected by zinc chelation with TPEN (Fig. 6d), suggesting that in addition to the proposed TLR-zinc signaling axis, another pathway also contributed to the degradation of sortilin by TLR signals. Therefore, as well as PCBP1, other factor(s) may also be involved in these aspects of sortilin regulation. One possible candidate for targeting sortilin mRNA is the endoribonuclease Regnase-1, which recognizes stem-loop structures in target mRNAs and then destabilizes the mRNAs^11^. Mino and colleagues have recently identified various targets of Roquin and Regnase-1 by RIP-seq analysis^38^. It is noteworthy that this RIP-seq analysis indicated that no sortilin-encoding mRNA was enriched by co-precipitation with either Regnase-1 or Roquin. These results are reasonable, however: Regnase-1 is active in resting cells to limit the basal transcription of target genes^39^, whereas sortilin is expressed in resting cells (Figs 1 and 4), indicating that Regnase-1 does not target sortilin mRNA. TLR-downstream molecules other than Regnase-1 and Roquin are therefore likely to be involved in destabilization and degradation of sortilin mRNA. Thus, further experiments are required to uncover the mechanistic details of how sortilin mRNA is controlled by TLR signals. Collectively, our findings underscore the importance of the posttranscriptional regulation of sortilin by PCBP1 in the posttranslational modulation of cytokine production in immune cells.

## Methods

### Mice

C57BL/6 mice (female, 6–7 weeks old) were purchased from Sankyo Labo Service Corporation (Tokyo, Japan). All animal experimental protocols were approved by the Animal Research Committee of the Medical Research Institute, Kanazawa Medical University. The methods were carried out in accordance with the approved guidelines.

### Cell culture and reagents

All cells were cultured at 37 °C in a humidified 5% CO_2_ atmosphere. Medium composition for primary culture is described below. RAW 264.7 cells and HEK293T cells were grown in DMEM (Wako Chem., Osaka, Japan) supplemented with 10% FCS (Sigma-Aldrich, St Louis, MO), 100 U/ml penicillin, and 100 U/ml streptomycin (Wako Chem.). The TLR ligands poly I:C (Imgenex, San Diego, CA), LPS (Sigma-Aldrich), imiquimod (Imgenex), CpG-A (1585: Nihon Gene Research Laboratories, Sendai, Japan) and CpG-B (1668: Nihon Gene Research Laboratories) were used for TLR stimulation. N,N,N’,N’-tetrakis-(2-pyridylmethyl)ethylenediamine (TPEN; Sigma-Aldrich) was used for zinc chelation, and zinc loading of cells was performed by the addition of zinc sulfate (Sigma-Aldrich) and the ionophore pyrithione (Sigma-Aldrich). Doxycycline hydrochloride (Wako Chem.) was used at 1 μg/ml for the pTet-off system.

### Preparation of bone marrow-derived immune cells

Bone marrow was isolated from the femurs of C57BL/6 mice. For plasmacytoid dendritic cell culture, 2 × 10^6^ cells/ml were seeded in RPMI 1640 without L-glutamine (Sigma-Aldrich) containing 10% FCS, GlutaMAX Supplement (Life Technologies, Gaithersburg, MD), 100 U/ml penicillin-streptomycin (Wako Chem.), 100 μM sodium pyruvate (Wako Chem.), MEM non-essential amino acids solution (Wako Chem.) and 100 ng/ml Flt3-L (Miltenyi Biotec, Auburn, CA). On day 4, fresh culture medium was supplemented. On day 8, experiments were performed after isolation of B220^+^ cells as pDCs by magnetic cell sorting with MACS separation columns (Miltenyi Biotec) following incubation with anti-B220-conjugated MACS microbeads (Miltenyi Biotec). Preparation of conventional dendritic cells was carried out as previously described^40^,^41^,^42^. On day 10, we used nonadherent cells for the experiments. Mast cells and eosinophils were prepared as previously described^43^,^44^. Preparation of basophils was carried out as previously described^45^ with minor modifications. On day 12, experiments were performed after isolation of c-kit^−^ DX5^+^ cells as basophils by magnetic cell sorting with MACS separation columns (Miltenyi Biotec) following incubation with anti-c-kit- or anti-DX5-conjugated MACS microbeads (Miltenyi Biotec).

### Preparation of mouse peritoneal macrophages and splenic CD4^+^, CD8^+^ and B220^+^ cells

Peritoneal exudate cells (PECs) were collected with 6 ml of ice-cold PBS from C57BL/6 mice and resuspended in RPMI 1640 medium containing 10% FCS, GlutaMAX Supplement and 100 U/ml penicillin-streptomycin. PECs were cultured in plastic plates for 2 h and monolayer cells were harvested as peritoneal macrophages. CD4^+^, CD8^+^ and B220^+^ splenocytes were isolated from spleens of C57BL/6 mice by magnetic cell sorting with MACS separation columns (Miltenyi Biotec). Anti-CD4-, -CD8- and -B220-conjugated MACS microbeads (Miltenyi Biotec) were used to isolate CD4^+^, CD8^+^ and B220^+^ splenocytes, respectively.

### Plasmid construction and transfection

Plasmids containing full-length sortilin cDNA and IFNA2 cDNA were purchased from DNAFORM (Yokohama, Japan). Open reading frames corresponding to sortilin and IFNA2 were amplified from these plasmids and cloned into pEGFP-N3 and pmCherry-N1 (Clontech, Palo Alto, CA), respectively. IFNA2 coding sequence lacking the N-terminal signal sequence was chemically synthesized by Eurofins Genomics (Tokyo, Japan) and cloned into pGEX-6P-1 (GE Lifesciences, Piscataway, NJ). A CRE-containing sequence in the 3′ UTR of sortilin mRNA (500 bp) was amplified from a plasmid containing sortilin cDNA and subcloned into pTRE2 (Clontech, Palo Alto, CA). CRE deletion mutants were generated with pTRE2-CRE as a template using a PrimeSTAR Mutagenesis Basal Kit (Takara Bio Inc., Otsu, Japan) according to the manufacturer’s instructions. Plasmid transfection was carried out with ScreenFect A (Wako Chem.) according to the manufacturer’s instructions. Primers used for plasmid construction are shown in Supplementary Table I.

### Protein depletion by siRNA and immunoblot analysis

Sortilin depletion in pDCs was carried out by nucleofection of siRNA (siGENOME SMARTpool, Mouse SORT1 siRNA; GE Dharmacon, Lafayette, CO) using a Nucleofector 2b with a Mouse Dendritic Cell Nucleofector Kit (Lonza, Basel, Switzerland) according to the manufacturer’s instructions. PCBP1 depletion in RAW264.7 cells was carried out by transfection of siRNA (siGENOME SMARTpool Mouse Pcbp1 siRNA; GE Dharmacon) using ScreenFect siRNA (Wako Chem.) according to the manufacturer’s instructions. A non-targeting sequence siRNA pool (siGENOME Non-Targeting siRNA Pool #2: GE Dharmacon) was used as a control.

Whole cell lysates were prepared with lysis buffer (100 mM Tris-HCl (pH 8.0), 1% Nonidet P-40, 1 mM DTT, Halt protease inhibitor cocktail, EDTA-free (Thermo Fisher Scientific, Waltham, MA) and 50 mM KCl), followed by centrifugation at 4 °C. Protein was quantified using a Pierce BCA Protein Assay Kit (Thermo Fisher Scientific) with BSA as standard. Protein samples to be examined by immunoblotting were separated by SDS-PAGE and electroblotted onto either a polyvinylidene difluoride membrane filter (Merck Millipore, Billerica, MA) for chemiluminescence detection or a nitrocellulose membrane filter (BioRad Laboratories, Richmond, CA) for the detection of infrared fluorescence. The filter was incubated with anti-PCBP1 (1:10,000)^17^, anti-sortilin (R&D; 1:1,000) and anti-actin (Sigma-Aldrich; 1:10,000) antibodies. Enhanced chemiluminescence detection was carried out using Clarity Western ECL Substrate (BioRad Laboratories) for the detection of secondary antibodies conjugated with horseradish peroxidase. Chemiluminescence images were obtained with an LAS-4000 mini imaging system (GE Lifesciences). The Odyssey system (LI-COR Biosciences, Lincoln, NE) was used to detect infrared dyes conjugated to secondary antibodies.

### Surface plasmon resonance analysis of sortilin

Recombinant proteins of mouse sortilin and cytokines except for mouse IFN-α were purchased from R&D. GST-fused mouse IFN-α was purified as follows: the plasmid pGEX-IFNA2 was introduced into *Escherichia coli* strain Rosetta 2(DE3) (Novagen, Madison, WI). Plasmid-bearing cells were initially grown aerobically at 37 °C on L medium^46^ containing 100 μg/ml ampicillin. Expression of recombinant proteins was then induced by the addition of 0.2 mM IPTG at 20 °C for 18 h. Cell lysates were prepared with FastBreak Cell Lysis Reagent (Promega, Madison, WI). Isolated supernatants were purified with a GSTrap 4B column (GE Lifesciences). Eluted protein fractions were then desalted through a HiTrap Desalting column (GE Lifesciences) and fractions containing purified proteins were concentrated with Vivaspin 20 spin columns (GE Lifesciences). Concentrated proteins were stored at −80 °C until use. Surface plasmon resonance analysis was performed as described^47^ on a Biacore X instrument (GE Lifesciences). Kinetic parameters were determined using BIAevaluation software.

### Confocal microscopy

For fluorescent protein detection, HEK293T cells that transiently expressed IFNA2-mCherry were used. Transfection of pEGFP-Sortilin and pmCherry-IFNA2 was carried out with ScreenFect A (Wako Chem.). After an 18-h incubation, cells were fixed in 4% paraformaldehyde in PBS for 10 min, and then washed three times in PBS. To observe co-localization of endogenous sortilin with endogenous IFN-α by indirect immunofluorescence, pDCs were stimulated with 3 μM of CpG-A (Nihon Gene Research Laboratories) in culture medium for induction of endogenous IFN-α. After a 6 h incubation, cells were fixed in 4% paraformaldehyde in PBS for 10 min, and then washed three times in PBS. Specimens were incubated for 5 min with PBS containing 0.5% Triton X-100, followed by a 30 minute incubation with 1% bovine serum albumin in PBS. The primary antibodies [anti-sortilin (R&D; 1:100); anti-IFN-α (Pestka Biomedical Laboratories, New Brunswick, NJ; 1:100); anti-syntaxin 6 (Cell Signaling Technology, Beverly, MA; 1:100)] and the secondary antibodies [DyLight 488-conjugated donkey-anti-goat IgG (Rockland, Gilbertsville, PA; 1:100); DyLight 649-conjugated donkey-anti-rabbit IgG (Rockland; 1:100)] were diluted in PBS and incubated for 18 h and 1 h, respectively. Specimens were mounted with ProLong Diamond Antifade Mountant (Thermo Fisher Scientific). Images were obtained with a Carl Zeiss LSM 710 laser scanning microscope (Carl Zeiss, Oberkochen, Germany) and analyzed with ZEN 2010 software (Carl Zeiss). The linescan analysis was performed with ZEN2010 software (Carl Zeiss). 30 cells were randomly selected and the Pearson’s correlation coefficient for each cell was determined with ZEN2010 software (Carl Zeiss).

### Quantitative real time PCR

Total RNA from 1−5 × 10^5^ cells was isolated with ReliaPrep RNA Cell Miniprep System (Promega) according to the manufacturer’s instructions. For isolation of total RNA from mouse tissues, dissected tissue samples (about 5 mg) were immersed immediately in RNAlater RNA Stabilization Reagent (QIAGEN, Hilden, Germany). Subsequently, the total RNA was purified with an miRNeasy Mini Kit (QIAGEN) according to the manufacturer’s instructions. cDNA was synthesized using 100 ng of total RNA with an iScript Advanced cDNA Synthesis Kit (BioRad Laboratories) or 500 ng of total RNA with ReverTra Ace qPCR RT Master Mix (Toyobo, Ohtsu, Japan), according to the manufacturers’ instructions. Quantitative real-time PCR was performed on the DNA Engine Opticon 2 system (BioRad Laboratories) with GoTaq qPCR Master Mix (Promega). Cycle threshold values were normalized to the housekeeping gene GAPDH. Primers for qRT-PCR are shown in Supplementary Table I.

### Electrophoretic mobility shift assay (EMSA)

Whole cell lysate from macrophages was prepared with lysis buffer (100 mM Tris-HCl (pH 8.0), 1% Nonidet P-40, 1 mM DTT, 50 mM KCl and protease inhibitor cocktail EDTA-free (Sigma-Aldrich)), followed by centrifugation at 4 °C. IR800 dye-conjugated oligo RNA (WT CRE; 5′-CAUGUGGCCCCUCCCCCGGCUGU-3′) was synthesized by IDT (Coralville, IA). Unlabeled oligo RNAs were synthesized by HSS (Sapporo, Japan). Assays contained 4 μg of whole cell lysate and IR800 dye-conjugated poly-C probe (200 nM) in 20 μl of binding buffer (50 mM Tris-HCl (pH 7.4), 150 mM NaCl, 100 U/ml RNasin plus Ribonuclease Inhibitor (Promega)) with or without unlabeled WT CRE probe (10 μM) as specific competitor or 10 μM nonspecific competitor, which were introduced by disruption of the CRE with a series of base substitutions (mt CRE; 5′-CAUGUGGAAAAUAAAAAGGCUGU-3′). Binding reactions were performed on ice for 30 min. Antibody supershift experiments were performed with 1 μg of anti-PCBP1 antibody (MBL, Nagoya, Japan) on ice for 5 min after the RNA/protein binding reaction. Normal rabbit IgG (Merck Millipore) was used as a negative control. Samples were separated on 6% polyacrylamide gels (Wako Chem.) and analyzed by the Odyssey system.

EMSA with a yeast pep4Δ strain transformed with pYES2 FLAG-PCBP1 was performed as described^17^. Ferrous ammonium sulfate and zinc sulfate (both 100 μM) were incubated with cell lysate prior to incubation with the probes.

### RNA immunoprecipitation and RIP-seq analysis

Whole cell lysate for RNA immunoprecipitation was prepared from 2 × 10^7^ macrophages using a RiboCluster Profiler RIP-Assay kit (MBL) according to the manufacturer’s instructions, with minor modifications. Sixty microliters of Magnetic Beads ProteinA/G (Merck Millipore) bound to 15 μg of anti-PCBP1 (MBL) or rabbit IgG (Merck Millipore) was incubated with lysate at 4 °C for 18 h. RNA precipitated with antibodies was purified with an miRNeasy Mini Kit (QIAGEN) and used for RIP-seq and RT-PCR analysis. For RT-PCR, cDNA was synthesized with an iScript Advanced cDNA Synthesis Kit (BioRad Laboratories). PCR was carried out using a GoTaq Master Mix (Promega) with the primers shown in Supplementary Table I. For RIP-seq analysis, poly-A-containing RIP products were purified using poly-T oligo-conjugated magnetic beads. RNA libraries were prepared with NEBNext Ultra Directional RNA Library Prep Kit for Illumina (New England Biolabs, Ipswich, MA) according to the manufacturer’s instructions. The libraries for RIP and input samples were subjected to sequencing on HiSeq 1500 (Illumina, San Diego, CA) with 51-bp single-end reads.

### RIP-seq data processing

Low-quality reads were discarded using the FASTQ Quality Filter program in FASTX-Toolkit version 0.0.13 (http://hannonlab.cshl.edu/fastx_toolkit/index.html) based on averaged Phred quality scores throughout the entire length (quality score of <30). The remaining reads were mapped using the Bowtie2 program^48^ version 2.2.4 with ‘-a’ and ‘-nofw’ options on the transcript sequences constructed from the reference mouse genome (UCSC mm10). Duplicate reads were excluded from the mapped reads using the SAMtools program^49^ version 0.1.19, and only non-duplicate reads were kept for further analysis. To identify peaks and regions of PCBP1 enrichment, the reads were analyzed using the MACS program^50^ version 2.0.19 with the options ‘–nomodel’ and ‘–to-large’. The threshold *q*-value for enriched regions of PCBP1 was set to 10^−10^. The input reads were used to normalize the background noise.

### Motif analysis

For PCBP1 peak regions, we conducted further analysis with the motif discovery program, DREME^51^ version 4.10.0, in the MEME Suite^52^, with the following options: -min 6 -max 12 -norc -e 1e-10. Background sequences for DNA motif detection were generated from a set of DNA sequences with the same nucleotide composition as the peak regions. Discovered motifs were also searched for PCBP1 peak regions by the MAST^53^ program, version 4.10.0, in the MEME Suite^52^, with the option ‘-mt 1e-5′.

### Statistical analysis

Statistical analysis was carried out by ANOVA using GraphPad Prism software. One-way and two-way ANOVA were used for experiments with one and two variables, respectively. A p value of less than 0.05 was considered statistically significant.

### Data deposition

All data have been deposited at DDBJ. Accession number is DRA004185 for the data in Supplementary Table II.

## Additional Information

**How to cite this article**: Yabe-Wada, T. *et al.* TLR signals posttranscriptionally regulate the cytokine trafficking mediator sortilin. *Sci. Rep.* **6**, 26566; doi: 10.1038/srep26566 (2016).

## Supplementary Material

Supplementary Information

Supplementary Dataset
