# Supplementary material for: TLR signals posttranscriptionally regulate the cytokine trafficking mediator sortilin
Source: Sci Rep. 2016 May 25;6:26566. doi: 10.1038/srep26566 (PMC4879639; doi:10.1038/srep26566)
Supplement: Supplementary Information [file srep26566-s1.pdf]

Supplementary Information

TLR signals posttranscriptionally regulate the cytokine trafficking mediator sortilin

Toshiki Yabe-Wada, Shintaro Matsuba, Kazuya Takeda, Tetsuya Sato, Mikita Suyama, Yasuyuki Ohkawa, Toshiyuki Takai, Haifeng Shi, Caroline C. Philpott, Akira Nakamura

Supplementary Information includes:

- Supplementary Table I. Primer sequences used in this work
- Supplementary Table II. RIP-seq Data from PCBP1 IP Experiments, Related to Figure 5.

Supplementary Table I: Primer sequences used in this work

Gene	Sequence	Use	Source
Gapdh	CATGGCCTTCCGTGTTCCTA<br>CCTGCTTCACCACCTTCTTGAT	qRT-PCR	Ref. 54
Sortilin	ACTTCACTGGGCTTGCTTCC<br>CCTCTTCACAATTCCGCTCA	qRT-PCR	This study
	TGCCTGTTGTCCTCCCCTCT<br>TGCAGTGGTGGTCTTGATCCT	RIP/RT-PCR<br>qRT-PCR	This study
	GCATGTGGGGCTGTGAGGGGCAGCCG<br>CCAGGCGGCCGCACACAATGCTAAA	Construction for<br>pTRE2-CRE	This study
IFNA1	GCCTTGACACTCCTGGTACAAATGAG<br>CAGCACATTGGCAGAGGAAGACAG	qRT-PCR	This study
PCBP1	CACGTAACGAGCCCAACTCC<br>CACCGACTCCCCTTTCTTCC	qRT-PCR	This study

References

54 Tsujita, Y. *et al*. Nuclear targeting of Akt antagonizes aspects of cardiomyocyte hypertrophy. *Proc Natl Acad Sci USA* **103,** 11946-11951, (2006).
